# Rapid Emergency Department Physical Space Modifications for COVID-19: Keeping Patients and Health Care Workers Safe

**DOI:** 10.1017/dmp.2021.248

**Published:** 2021-08-04

**Authors:** Emily G Wessling, Amanda H Randolph, Luke A Neill, Kumar R Gandhi, Michael Conrardy, Jason D Chodakowski, Matthew Kippenhan, Timothy M Loftus, Sanjeev Malik

**Affiliations:** Department of Emergency Medicine, Northwestern University Feinberg School of Medicine, Chicago, IL, USA

**Keywords:** covid-19, emergency medicine, emergency department operations, disaster planning, emergency care systems, efficiency

## Abstract

The COVID-19 pandemic has placed significant strain on emergency departments (EDs) that were not designed to care for many patients who may be highly contagious. This report outlines how a busy urban ED was adapted to prepare for COVID-19 via 3 primary interventions: (1) creating an open-air care space in the ambulance bay to cohort, triage, and rapidly test patients with suspected COVID-19, (2) quickly constructing temporary doors on all open treatment rooms, and (3) adapting and expanding the waiting room. This description serves as a model by which other EDs can repurpose their own care spaces to help ensure safety of their patients and health care workers.

## Introduction

The novel coronavirus disease-2019 (COVID-19) has become a once-in-a-generation pandemic, swiftly infecting millions across the world. This pandemic has placed significant pressure on the global health care workforce, as it stresses health system capacity and increases the risk of infection among health care workers.^1^ Early data from Wuhan, China showed that 29% of cases were health care workers.^2^ As cases and knowledge of the disease spread, adoption of infection control measures and personal protective equipment (PPE) helped to successfully decrease health care worker cases in China to 3.8%.^3^

While PPEs are critical to protecting the health care workforce, engineering controls also play a significant role in reducing occupational exposure rates.^4^ Historically, emergency departments (EDs) throughout the world have adapted physical spaces to better accommodate pandemic populations. During influenza outbreaks, several hospitals such as Texas Children’s Hospital (Houston, Texas, USA) created drive-through clinics to minimize exposures and increase throughput.^5^ In response to COVID-19, South Korea optimized drive-up clinics to rapidly test patients, thereby minimizing exposure risk, decreasing PPE utilization, and using personal vehicles as a means of expanding isolation rooms.^6^ In other areas, health care workers looked to modify intrinsic ED structures. In Italy, researchers created a checklist for how to expand ED capacity to accommodate pandemic surge patients, calling for dedicated waiting areas and pathways for COVID-19 patient movement through the ED.^7^ Also, health care professionals in a German acute care clinic took precautions and set up a 3-point entry system with a pre-triage screening point to prevent intra-hospital contamination.^8^

Center for Disease Control and Prevention (CDC) guidelines and studies on infection prevention from Italy suggest patient care should occur in negative airflow or neutral airflow rooms.^9,10^ Many busy EDs utilize hallway spaces and vertical flow models to address high patient volumes and in-patient boarding. However, these approaches often do not adhere to these guidelines, forcing many EDs to rapidly redesign spaces that have become unsafe during a pandemic.

This intervention occurred in an urban academic ED (> 94000 annual visits) in Chicago, Illinois, USA. This ED has many open treatment rooms with curtain barriers rather than closed doors, and patients are often pulled to the hallway to create additional capacity during times of crowding. In response to COVID-19, rapid solutions were developed to increase the number of neutral airflow rooms in the department while reducing hallway use. In order to minimize risk of transmission, the ED was adapted via 3 primary interventions: (1) creating an open-air care space in the ambulance bay to cohort, triage, and rapidly test patients with suspected COVID-19, (2) constructing temporary doors on all open treatment rooms, and (3) adapting and expanding the waiting room.

## Exterior ED Expansion: Temporary Open-Air Care Space

In the 2003 SARS outbreak, inefficient ventilation contributed to increased viral spread.^11^ In response, hospitals in Hong Kong utilized natural ventilation in patient wards.^12^ Building on this knowledge, temporary open-air care spaces were created by repurposing the ambulance bay (Image 1A). This additional space facilitated patient grouping, improved ventilation, and increased patient distancing to minimize disease transmission risk.

**Figure 1.**
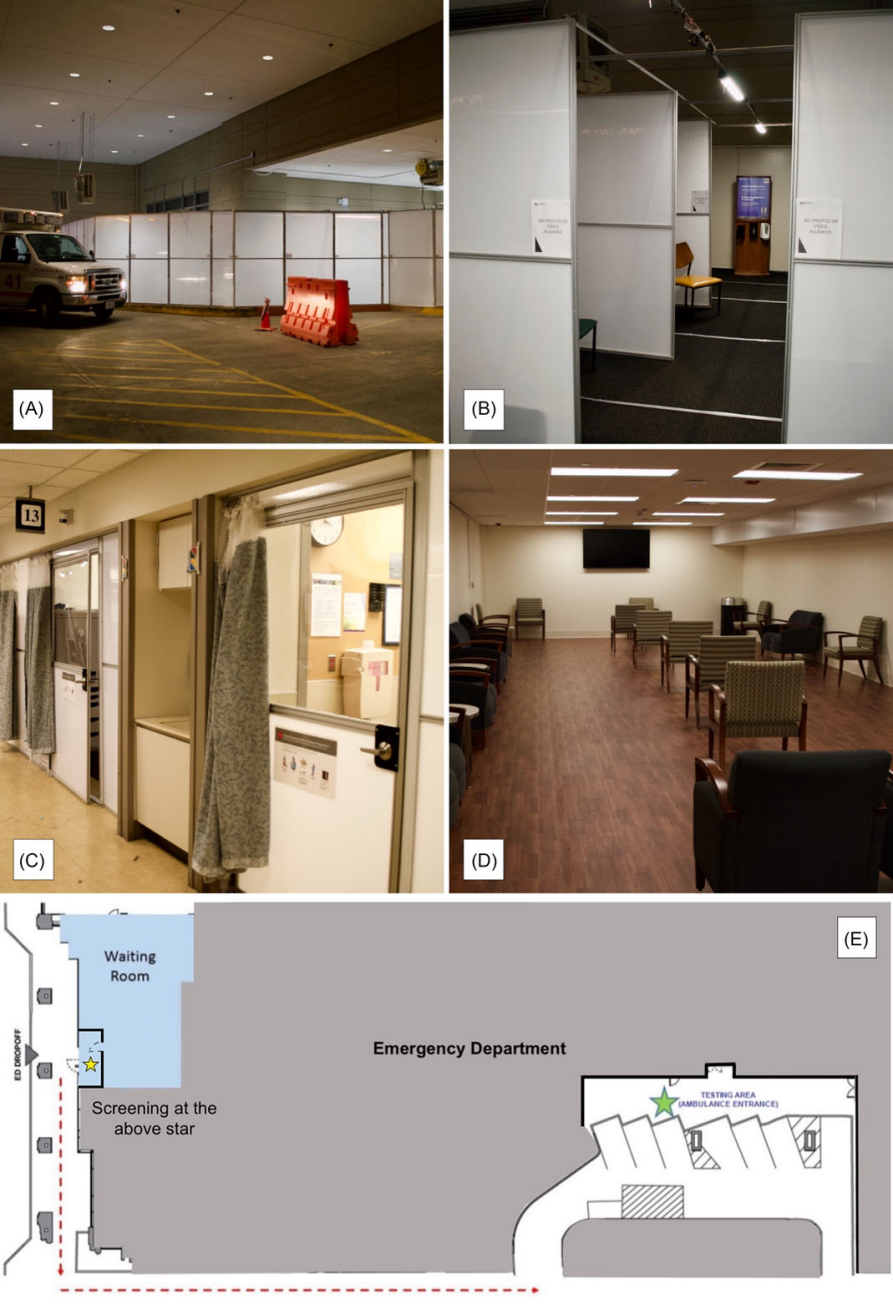
Physical space modifications in response to COVID. 1A–1B: External and internal depiction of temporary open-air care spaces in ED ambulance bay. 1C: temporary construction panels added to existing curtained ED treatment rooms. 1D: secondary negative airflow annex ED waiting room repurposed from unused facility space. 1E: schematic of patient flow from immediate screening at ED waiting room entrance to open-air care space.

Using the ambulance bay as an open-air care space obviated the need for ventilation construction. To ensure safety while maintaining open airflow, the ambulance bay was partitioned by repurposing commercially available temporary construction panels (**Image 1B**). Each partitioned care space was equipped with documentation computers, testing supplies and sanitation materials, and PPE donning and doffing stations were installed at the staff entrance. The overall construction of this care space occurred in less than 4 days, costing approximately $75000. Due to the nature of the materials used for the creation of this space, this care space can be easily constructed and deconstructed as needed. Staffing costs were similar to other areas of the ED, but overall costs were partially mitigated by staff restructuring within other care spaces.

Physicians, advanced practice providers, nurses, and ED assistants all staff this care space. They don PPE and enter the open-air care space via a separate staff hallway. All patients presenting to the ED (approximately 250 patients per day) were screened for potential treatment in this care space. Well-appearing patients suspected to have COVID-19 are identified at the waiting room entrance, masked, and walked outside to the care space. This outdoor walkway prevents these patients from moving through the ED. Upon assessment in the open-air care space, testing for COVID-19 and/or chest radiography is performed as indicated, and the patient is dispositioned. Patients who require further evaluation and potential admission enter through the ambulance bay, where they are immediately placed in a closed-door room (**Image 1E**). Patients were often discharged prior to the results of their COVID-19 test, and results were called by a designated follow up health care team.

The creation of an open-air care space has successfully decreased stress on the existing ED. Over 30 days, this space was able to treat over 900 total patients (˜ 30 patients per day), which represented 15% of daily volumes. At peak volumes, over 70 patients were seen between 10 AM and 8 PM, the operational hours of this space. Overall, this space increased ED capacity while maintaining compliance with infection control guidelines, consolidating PPE, and improving care efficiency for low acuity patients with suspected COVID-19.

## Creating Additional Neutral Airflow Rooms

It was infeasible to convert the entire ED to negative airflow rooms, therefore, all curtained open treatment rooms were retrofitted with temporary construction panels with doors costing approximately $20000 (**Image 1C**). This provided a physical barrier between providers and patients, creating neutral airflow rooms. These doors included a clear window, facilitating patient monitoring while maintaining appropriate isolation. Each door took less than 2 hours to attach, and the ED was refitted in less than 1 day. These installations increased neutral airflow room capacity by greater than 35% without disrupting the operational flow of the ED during construction.

## Addition of Secondary Waiting Room: Annex and Chairs

The existing negative airflow waiting room was re-designed to comply with current CDC guidelines of 6 feet of separation. However, this redesign decreased waiting room capacity substantially (from 30 to 12) necessitating the creation of a second negative airflow annex waiting room. This occurred through the conversion of dead-space in the facility not adjacent to the ED over 1-week costing approximately $150000 (**Image 1D**). While the location for this annex waiting area is suboptimal and will require additional staffing, the benefits from an infection control perspective made this an intervention worth pursuing. Should volumes increase beyond the capacity allotted by the enclosed rooms, these waiting rooms can be split into care spaces.

## Conclusion

Through these 3 physical space modifications, ED and non-ED spaces were successfully repurposed to safely and efficiently evaluate patients with suspected COVID-19. The open-air care space helped increase the ED’s ability to see patients with lower-acuity respiratory complaints in an efficient manner. The addition of the temporary doors to the curtained care spaces broadened their utility and facilitated efficient placement of higher acuity patients in more care spaces. The only drawback noted was based on the design of the doors themselves. The temporary doors selected had a threshold as part of their design, which made maneuvering equipment, such as ultrasound machines into the rooms, more challenging. Overall, their utility greatly outweighed the drawbacks. The annex waiting room added much needed capacity. These successful interventions were the design of an interprofessional team consisting of physicians, nurses, and members of the administration and operations teams. These structural changes were enabled, in part, by being part of a large health care system with the financial resources and ongoing construction partnerships to quickly enact these changes. However, although we as authors of this commentary acknowledge that such changes may not be financially or logistically viable in all settings, we offer this description as a model by which other EDs can repurpose their own care spaces to help ensure safety of their patients and health care workers.
